# β-Sitosterol Suppresses Lipopolysaccharide-Induced Inflammation and Lipogenesis Disorder in Bovine Mammary Epithelial Cells

**DOI:** 10.3390/ijms241914644

**Published:** 2023-09-27

**Authors:** Yating Fan, Jinglin Shen, Xinlu Liu, Junhao Cui, Jiayi Liu, Dongqiao Peng, Yongcheng Jin

**Affiliations:** Jilin Provincial Key Laboratory of Livestock and Poultry Feed and Feeding in Northeastern Frigid Area, Department of Animal Science, College of Animal Science, Jilin University, Changchun 130062, China; 18800439298@163.com (Y.F.); shenjinglinshen@aliyun.com (J.S.); liuxinlu629@163.com (X.L.); jhcui51@163.com (J.C.); loki980125@163.com (J.L.)

**Keywords:** β-sitosterol, HIF-1α signaling pathway, LPS, mastitis, mTOR signaling pathway

## Abstract

β-sitosterol, a natural plant steroid, has been shown to promote anti-inflammatory and antioxidant activities in the body. In this study, β-sitosterol was used to protect against lipopolysaccharide (LPS)-induced cell damage in bovine mammary epithelial cells, which are commonly studied as a cell model of mammary inflammatory response and lipogenesis. Results showed that treatment with a combination of LPS and β-sitosterol significantly reduced oxidative stress and inflammation, while increasing the expression of anti-apoptotic proteins and activating the hypoxia-inducible factor-1(HIF-1α)/mammalian target of rapamycin(mTOR) signaling pathway to inhibit apoptosis and improve lipid synthesis-related gene expression. Our finding suggests that β-sitosterol has the potential to alleviate inflammation in the mammary gland.

## 1. Introduction

Breast milk, as a source of nutrition for newborns, provides a variety of energy and nutrients. Supplementing breast milk during the first few months after birth can improve the immunity and intelligence of newborns, and reduce the occurrence of sudden infant death syndrome (SIDS) [1,2], Additionally, milk powder is also an important source of nutrients for infant growth and development during subsequent stages of growth. The mammary gland, the primary component of the human and mammalian breast, has a high morbidity rate. Once lesions occur, mammary inflammation can significantly impair the quality and production of breast milk, and it can also negatively affect the health of both the mother and the baby [3]. Clinical studies have shown that external factors are lipopolysaccharides (LPS) produced in vivo, considered to be an important stimulus to induce acute mammary inflammatory response [4]. As a major virulence factor of Gram-negative bacteria, a previous study reported that LPS induces an inflammatory response in cells through oxidative stress. Excessive oxidative stress will activate the nuclear factor-κB (NF-κB) signaling pathway, and then regulate the expression of inflammatory factors such as interleukin-1β (IL-1β), nuclear factor-κB p65 subunit (NF-κB p65), tumor necrosis factor-a (TNF-α) and cyclooxygenase-2 (COX2) [5]. In addition to inflammation, oxidative stress also induces apoptosis through the Caspase family pathway, reducing the number of mammary epithelial cells and ultimately leading to a decrease in milk production [6]. The current widespread use of antibiotics as a treatment for mastitis poses a health risk to both humans and animals due to the presence of drug residues and the development of antibiotic resistance in organisms. However, while biologic factor treatments and vaccine development show promise, they have not yet reached an ideal state. Therefore, it is urgent and crucial to screen for appropriate agents or drugs to prevent mastitis and alleviate its negative effects, which is important for the healthy development of women and female animals, as well as the growth and development of newborns.

Bovine mammary epithelial cells (MAC-T) are frequently used as a cellular model to investigate the mechanism of action in the mammary gland—for example, to investigate the mechanism of action of oils on the synthesis of milk lipids and milk proteins [7], the specific mechanism of inflammatory damage caused by virulence factors [8], and the absorption effect of antimicrobial peptides [9]. In our pervious study, we found that β-sitosterol could enhance the levels of milk protein and fat synthesis-related genes in MAC-T cells via regulating the expression of HIF-1α/mTOR signaling pathway [10]. β-sitosterol, as one of the most widely distributed phytosterols in the plant world, exists in the leaves, roots and fruits of many plants [11]. Since humans are unable to produce β-sitosterol, which is also the most consumed phytosterol in the body, so it is available for intake through the consumption of vegetable oils, fruits, vegetables, nuts, legumes, and other foods [12]. Generally, as a key active ingredient of safe and natural phytosterols, β-sitosterol has many pharmacological activities, such as anti-inflammatory, antioxidant, cholesterol-lowering, hypoglycemic and immunomodulatory, which has no genotoxicity or cytotoxicity to humans or animals [11,13]. In recent years, the anti-inflammatory and antioxidant effects of β-sitosterol have been the focus of research in the fields of clinical medicine and pharmacology. A previous study revealed that β-sitosterol had the potential to alleviate the fatty inflammatory response and associated with anti-insulin resistance and regulation of glucose metabolism by modulating the IκB kinase β (IKKβ)/NF-κB signaling pathway in a feeding experiment of type 2 diabetic mice [14]. Moreover, an alleviation of acute liver by β-sitosterol was found in mice when triggered by LPS-induced decrease in antioxidant enzyme activity [15]; meanwhile, β-sitosterol also diminished apoptosis by downregulating cysteaspase 3 (caspase 3) and cysteaspase 9 (caspase 9) protein expression and upregulating the protein expression of B-cell lymphoma-2 (Bcl-2), and restores NF-κB gene expression level in injured cardiomyocytes [16]. Thus, numerous studies demonstrated that β-sitosterol had been proved to exert positive anti-inflammatory, antioxidant effects in humans and animals; however, whether the same positive effect is observed for mammary inflammatory response and the exact underlying mechanisms are not yet clear.

Therefore, the specific purpose of this study was to investigate the effect of β-sitosterol on LPS-induced cell damage in bovine mammary epithelial cells, to determine whether it has a positive effect on milk lipid synthesis, and to elucidate the underlying protective mechanism involved. This study provides an experimental basis for understanding how β-sitosterol attenuates mammary inflammation-induced organismal damage.

## 2. Results

### 2.1. Effect of β-Sitosterol on the Viability of MAC-T Cells

**Figure 1:**
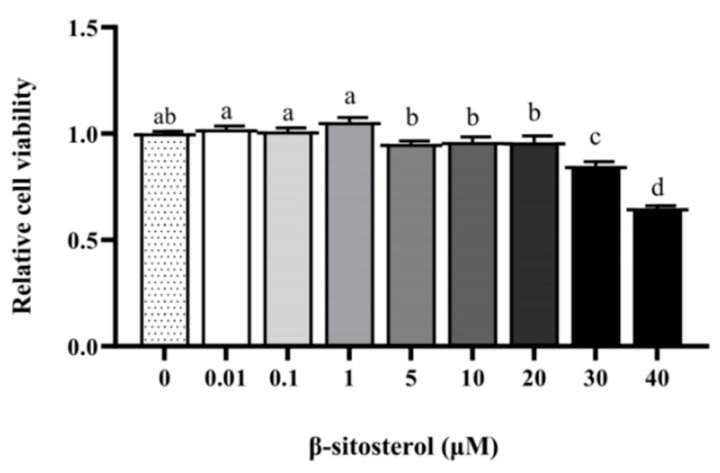
Effects of β-sitosterol on MAC-T cell viability. The cell viability of MAC-T cells treated with different concentrations of β-sitosterol (0, 0.01, 0.1, 1, 5, 10, 20, 30, and 40 μM) for 24 h. All values are presented as the mean ± SEM (n = 3). With the control group as 1, the treatment group was compared to it to calculate relative ratios. Different lowercase letters indicate significant differences compared to the control group (*p* < 0.05).

To investigate the effect of β-sitosterol on the viability of MAC-T cells, different concentrations of β-sitosterol (0, 0.01, 0.1, 1, 5, 10, 20, 30, and 40 μM) were treated in MAC-T cells. The result showed (Figure 1) that there was no significant difference among the 0.01–20 μM treatment groups compared to the control group (0 μM) (*p* > 0.05). Compared to the group treated with 1 μM β-sitosterol, cell viability significantly decreased with β-sitosterol concentrations exceeding 5 μM (*p* < 0.05). When the concentration of β-sitosterol was increased above 30 μM, the cell viability was significantly decreased (*p* < 0.05). We found that 1 μg/mL LPS could lead to cellular damage of MAC-T cells in our previous studies. Hence, based on the results from previous studies, we chose a combination of 1 μM β-sitosterol and 1 µg/mL LPS for subsequent cell treatments.

### 2.2. Effect of β-Sitosterol on LPS-Induced Oxidative Stress in MAC-T Cells

**Figure 2:**
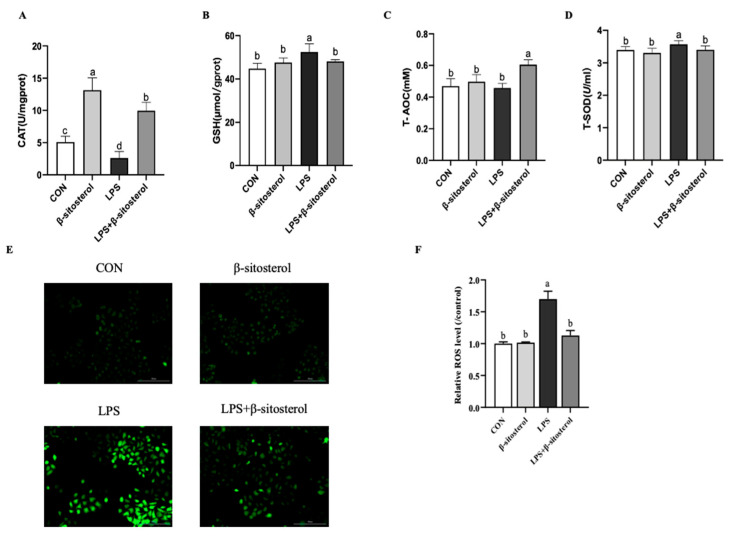
β-sitosterol alleviates LPS-induced oxidative stress in MAC-T cells. MAC-T cells were treated with β-sitosterol (1 μM) or LPS (1 μg/mL) for 24 h. (**A**) CAT activity in MAC-T cells. (**B**) GSH activity in MAC-T cells. (**C**) T-AOC activity in MAC-T cells. (**D**) T-SOD activity in MAC-T cells. (**E**) The control and treatment groups were stained with DCFH-DA, the cells were washed and examined by fluorescence microscopy. Scale bar = 200 μm. (**F**) The fluorescence intensity of ROS in MAC-T cells. All values are presented as the mean ± SEM (n = 3). Different lowercase letters indicate significant differences compared to the control group (*p <* 0.05).

The results showed that the LPS group significantly reduced the level of catalase (CAT) (*p* < 0.05, Figure 2A) and markedly enhanced the activity of glutathione (GSH) and total superoxide dismutase (T-SOD) compared with the control group in MAC-T cells, while this change was significantly restored in the LPS+β-sitosterol group (Figure 2B, *p* = 0.01, D, *p* = 0.033). The total antioxidant capacity (T-AOC) did not significantly change from the control group, while the level of T-AOC was dramatically increased by the addition of β-sitosterol (*p* < 0.001, Figure 2C). To detect the intracellular oxygen radical content, reactive oxygen species (ROS) was examined. The level of ROS was significantly elevated in LPS-induced MAC-T cells, while the LPS+β-sitosterol group recovered to the level of ROS in the control group (*p* < 0.05, Figure 2E,F).

### 2.3. Effect of β-Sitosterol on LPS-Induced Inflammatory Factors in MAC-T Cells

**Figure 3:**
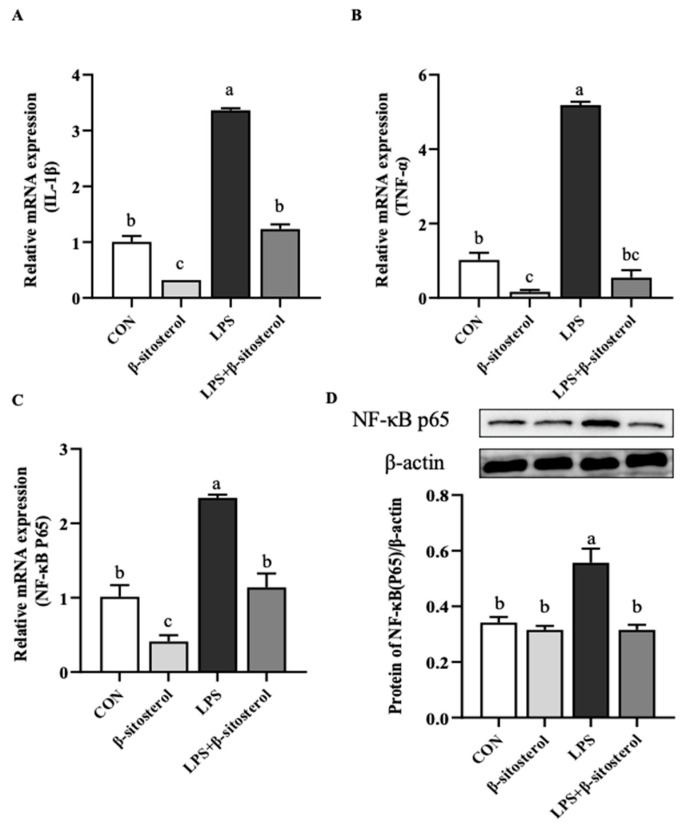
β-sitosterol attenuates LPS-induced inflammatory factor activity in MAC-T cells. MAC-T cells were treated with β-sitosterol (1 μM) or LPS (1 μg/mL) for 24 h. (**A**) IL-1β mRNA level in MAC-T cells. (**B**) TNF-α mRNA level in MAC-T cells. (**C**) NF-κB p65 mRNA level in MAC-T cells. (**D**) Protein level of NF-κB p65 in MAC-T cells. All values are presented as the mean ± SEM (n = 3). Different lowercase letters indicate significant differences compared to the control group (*p* < 0.05).

Compared with the control group, LPS significantly increased the mRNA expression levels of pro-inflammatory factors TNF-α and interleukin-1β (IL-1β), while the LPS+β-sitosterol group remarkably slowed them down (*p* < 0.05, Figure 3A,B). Furthermore, NF-κB p65, which is associated with inflammatory factor activation production, was detected by mRNA protein levels, resulting in the same changes as pro-inflammatory factor gene expression levels. β-sitosterol addition attenuated NF-κB p65 production by LPS-induced and restored it to the same level as the control group (*p* < 0.05, Figure 3C,D).

### 2.4. Effect of β-Sitosterol on LPS-Induced Apoptosis in MAC-T Cells

**Figure 4:**
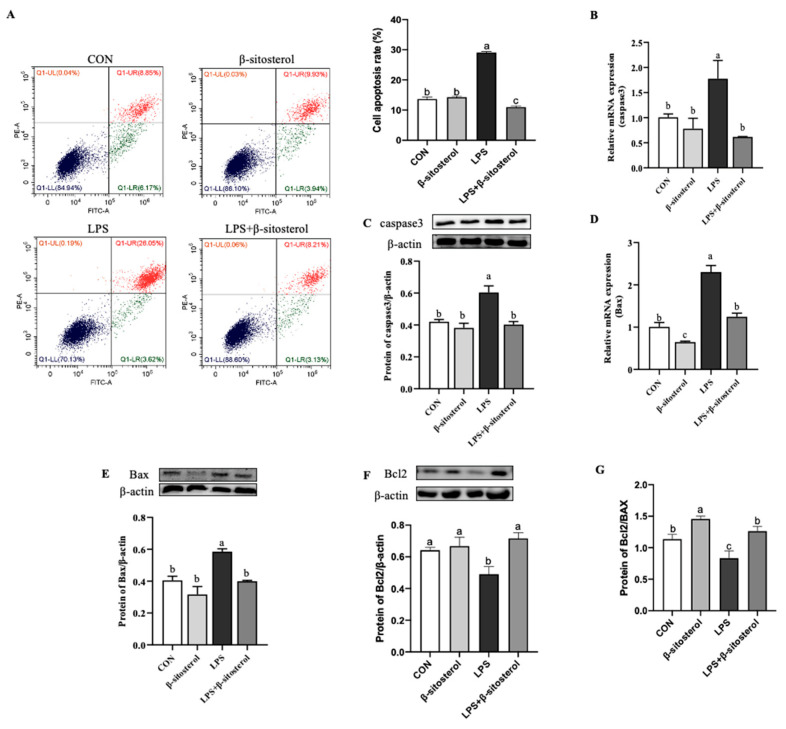
β-sitosterol protects LPS-induced apoptosis in MAC-T cells. MAC-T cells were treated with β-sitosterol (1 μM) or LPS (1 μg/mL) for 24 h. (**A**) The rate of apoptosis MAC-T cells was detected by flow cytometry. (**B**) Caspase 3 mRNA level in MAC-T cells. (**C**) Protein level of caspase 3 in MAC-T. (**D**) Bax mRNA level in MAC-T cells. (**E**) Protein level of Bax in MAC-T. (**F**) Protein level of Bcl-2 in MAC-T. (**G**) The ratio of Bcl-2 to Bax protein expression. All values are presented as the mean ± SEM (n = 3). Different lowercase letters indicate significant differences compared to the control group (*p* < 0.05).

According to the results of the MAC-T cells apoptosis rate by flow cytometry, it was shown that the apoptosis rate was significantly increased in LPS group compared with the control, but in the LPS+β-sitosterol group was found that β-sitosterol could greatly inhibit the apoptosis caused by LPS (*p* < 0.05, Figure 4A). The expression levels of mRNA and Protein of caspase 3 and pro-apoptotic protein B-cell lymphoma-2-associated X protein (Bax) was greatly raised in LPS group compared with the control group, and the expression of these pro-apoptosis was attenuated in the LPS+β-sitosterol group (*p* < 0.05, Figure 4B–E). However, the protein levels of Bcl-2 were remarkably reduced in LPS group compared with the control group and returned to the control levels after the addition of β-sitosterol (*p* = 0.02, Figure 4F). The Bcl-2/Bax radio was also significantly higher in the LPS+β-sitosterol group than the LPS group (*p* < 0.05, Figure 4G).

### 2.5. Effect of β-Sitosterol on LPS-Induced HIF-1α/mTOR Signaling Pathway in MAC-T Cells

**Figure 5:**
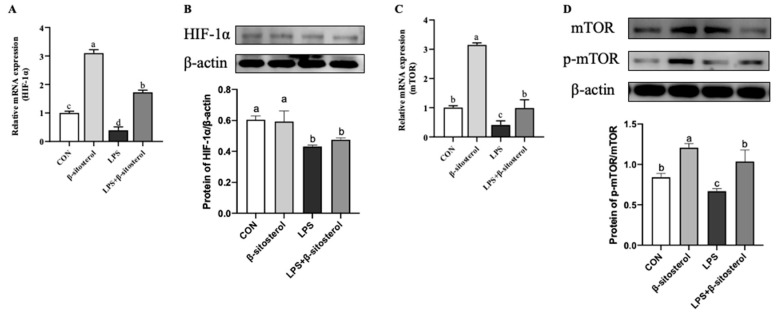
β-sitosterol restores LPS-induced HIF-1α/mTOR signaling pathway viability in MAC-T cells. MAC-T cells were treated with β-sitosterol (1 μM) or LPS (1 μg/mL) for 24 h. (**A**) HIF-1α mRNA level in MAC-T cells. (**B**) Protein level of HIF-1α in MAC-T cells. (**C**) mTOR mRNA level in MAC-T cells. (**D**) The ratio of p-mTOR to mTOR protein expression. All values are presented as the mean ± SEM (n = 3). Different lowercase letters indicate significant differences compared to the control group (*p* < 0.05).

Compared with the control group, the mRNA expression of HIF-1α and mTOR and HIF-1α protein expression were dramatically decreased in LPS group (*p* < 0.05, Figure 5A–D). In contrast, the mRNA levels of HIF-1α and mTOR were significantly upregulated in the LPS+β-sitosterol group compared with the LPS group (Figure 5A, *p* = 0.03, Figure 5C, *p* < 0.05), and the protein expression of HIF-1α was also remarkably raised (*p* < 0.05, Figure 5B). The ratio of p-mTOR/mTOR in the LPS group was remarkably lower than the control group and the β-sitosterol group; however, the addition of β-sitosterol recovers the level in the control group (*p* < 0.05, Figure 5D).

### 2.6. Effect of β-Sitosterol on LPS-Induced Fat Synthesis-Related Genes in MAC-T Cells

**Figure 6:**
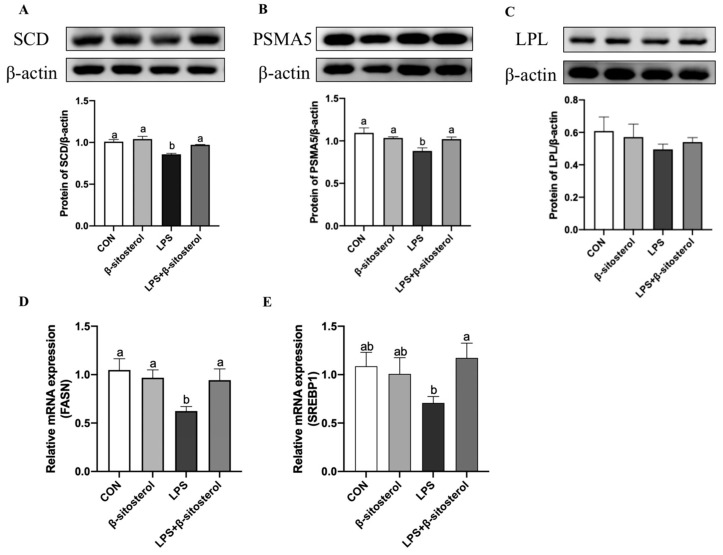
β-sitosterol promotes LPS-induced milk fat synthesis-related genes in MAC-T cells. MAC-T cells were treated with β-sitosterol (1 μM) or LPS (1 μg/mL) for 24 h. (**A**) Protein level of SCD in MAC-T cells. (**B**) Protein level of PSMA5 in MAC-T cells. (**C**) Protein level of LPL in MAC-T cells. (**D**) FASN mRNA level in MAC-T cells. (**E**) SREBP1 mRNA level in MAC-T cells. All values are presented as the mean ± SEM (n = 3). Different lowercase letters indicate significant differences compared to the control group (*p* < 0.05).

As for the fat synthesis, the protein expression of stearoyl coenzyme A dehydrogenase (SCD), proteasome 20 s subunit α5 (PSMA5) together with the mRNA levels of fatty acid synthase (FASN) and sterol regulatory element-binding protein 1 (SREBP1) were dramatically downgraded in the LPS group than the control group, while the levels of both in the LPS+β-sitosterol group were restored to the controls (Figure 6A,B*, p* = 0.03, Figure 6D,E*, p* < 0.05). Further, there was no significant effect of lipoprotein lipase (LPL) in the LPS group compared with the control group (*p* > 0.05, Figure 6C). 

## 3. Discussion

The mammary gland is an essential and highly prevalent component of the reproductive system in women and female animals. Mastitis, a condition caused by inflammation of the mammary gland, has serious negative impacts on mammary development, lactation, as well as physical and mental health in humans and livestock [17,18]. LPS, which originates from the outer wall of *E. coli*, infects mammary epithelial cells mainly through the gastrointestinal circulation. It is also considered a common inflammatory response mediator in mammary epithelial cells. Excessive metabolism of LPS in the cells leads to a significant increase in intracellular ROS levels and a decrease in cellular antioxidant capacity [19], which resulted in cellular inflammation, as well as affects the function and development of the mammary gland, reduces milk quality and production [20,21]. Therefore, it is crucial to alleviate the expression of inflammatory factors, relieve oxidative stress, and restore the expression of genes related to lipid synthesis as important strategies to prevent mammary inflammation.

As a natural phytosterol extracted from plants, β-sitosterol is mostly used in food and pharmaceutical industries. For instance, adding β-sitosterol to the diet of mice has been found to reduce the incidence of nonalcoholic fatty liver [22]. In addition, it has good effect in relieving colitis, prostate enlargement, and acute liver injury [23,24]. Therefore, we hypothesized that β-sitosterol has a similar effect in relieving mammary inflammation and discussed this issue. In the previous study of the group, we observed that β-sitosterol concentrations ranging from 0.1 to 10 μM positively influenced genes associated with milk fat and protein synthesis, with 1 μM showing the most pronounced effect [10]. In MAC-T cells, exposure to 1 μg/mL LPS typically resulted in cellular damage [25,26]. Therefore, we chose 1 μM β-sitosterol and 1 μg/mL LPS in the current study to investigate whether a protective effect against inflammation and oxidative stress in MAC-T cells.

Oxidative responses are critical for the organism, but an excessive oxidative reaction can damage tissues. The body has an inherent antioxidant defense mechanism that counteracts the harmful effects of oxidative stress. This defense mechanism operates in cells, effectively reducing the levels of ROS and mitigating the detrimental effects of oxidative stress. Additionally, β-sitosterol has antioxidant capacity and acts as a mild free radical scavenger, with the ability to restore LPS-induced changes in antioxidant enzyme levels [15]. The results of the present study indicate that β-sitosterol significantly reduced the LPS-induced elevation of ROS levels, which is consistent with previous findings showing that β-sitosterol can alleviate ROS levels in human keratinocytes [27]. CAT is a vital antioxidant enzyme that effectively eliminates accumulated ROS in the short term. Meanwhile, T-AOC serves as a marker for the total defense capacity of the body’s antioxidant system. GSH plays a crucial role in protecting enzymes and proteins from oxidation, while superoxide dismutase (SOD) converts the superoxide radical (O_2_^−^) into peroxide [28,29]. The addition of β-sitosterol significantly increased the reduced CAT activity and T-AOC levels induced by LPS. Furthermore, β-sitosterol also restored the increased intracellular GSH and T-SOD levels induced by LPS. These findings suggest that β-sitosterol may have a protective effect against LPS-induced oxidative damage in MAC-T cells.

Oxidative stress is closely related to inflammation, and in LPS-induced oxidative stress and inflammatory injury in macrophages, β-sitosterol inhibits the activation of the NF-κB signaling pathway by reducing the production of ROS, which in turn improves cell viability [30]. NF-κB is an important transcriptional regulator in cells that plays an essential role in the inflammatory response of mammary glands. Its activation leads to the expression of pro-inflammatory factors and results in an inflammatory response in cells. Our findings demonstrate that pretreatment with β-sitosterol significantly reduced the expression of pro-inflammatory factors and inhibited the activation of the NF-κB pathway in LPS-induced MAC-T cells. These results suggest that β-sitosterol has the potential to alleviate the LPS-induced inflammatory response in MAC-T cells.

Both ROS and the NF-κB signaling pathway play vital regulatory roles in apoptosis, which is the programmed cell death process that effectively removes damaged cells. Although there are multiple pathways that stimulate apoptosis, the subsequent apoptotic process is mainly regulated by the activation of the inactive intracellular caspase family [31]. As the apoptotic executor, caspase 3 activation marks the occurrence of apoptosis. Excessive ROS production triggers the activation of caspase 9, the initiating protease of the apoptotic vesicle complex, leading to upregulation of downstream caspase 3, which ultimately induces apoptosis [32]. When ROS accumulation is excessive, it also releases a variety of pro-apoptotic-related factors, such as Bax and Bak, by stimulating the Bcl-2 protein family to alter the permeability of the cell membrane, which further activates the Caspase family to induce apoptosis [33]. Our study found that pretreatment with β-sitosterol prevents LPS-induced apoptosis in MAC-T cells. We visualized this result using flow cytometry and measured the expression levels of genes and proteins in the Caspase/Bcl2 signaling pathway. Specifically, β-sitosterol significantly reduced the expression of pro-apoptotic genes while increased the expression of anti-apoptotic genes. Furthermore, β-sitosterol was able to recover LPS-induced apoptosis in MAC-T cells, as indicated by the Bcl-2/Bax ratio. Our result was consistent with the finding that β-sitosterol inhibits cardiomyocyte apoptosis by downregulating the gene expression of Caspase/Bcl-2 signaling pathway [16]. This suggested that β-sitosterol could alleviate apoptosis caused by LPS by inhibiting the Caspase/Bcl-2 signaling pathway.

The HIF-1α/mTOR signaling pathway has been shown to play an essential role in various aspects, including cell proliferation, immune regulation, and cell metabolism [34]. B-cell activating factors have the ability to attenuate LPS-induced inflammatory damage in mouse primary microglia through the HIF-1α/mTOR signaling pathway [35]. Activating the HIF-1α/mTOR signaling pathway can also bring about changes in cell cycle and apoptosis by generating downstream factor activation. In our study, we found that β-sitosterol enhanced the expression levels of mTOR and HIF-1α genes, restored the down-regulation of mTOR and HIF-1α genes stimulated by LPS to normal levels, and significantly elevated the p-mTOR/mTOR ratio upon its addition. These findings suggest that β-sitosterol may protect and alleviate inflammatory damage in mammary glands by counteracting the inhibitory effect of LPS on the HIF-1α/mTOR signaling pathway.

The HIF-1α/mTOR signaling pathway is a critical center for many pathways affecting upstream factors of milk protein and lipid synthesis-related genes [36]. As reported in our previous study, the addition of β-sitosterol to MAC-T cells had a positive effect on the signaling pathway involved in milk protein and fat synthesis [10]. As mentioned previously, mastitis in mammary gland leads to a decrease in milk quality, while milk fat is one of the key components of milk, and the HIF-1α/mTOR signaling pathway promotes the expression of FASN, which is a gene related to fatty acid synthesis from scratch, by regulating SREBP1, a key regulator of lipid synthesis-related gene transcription [37,38]. SCD, as a critical enzyme for monounsaturated fatty acid synthesis, is also involved in the regulation of SREBP1, which together with LPL regulates intracellular lipid metabolism [39]. PSMA5, an important enzyme for the synthesis of conjugated linoleic acid, is regulated by HIF-1α and is involved in the synthesis of milk lipids [40,41]. Our current study found that the addition of β-sitosterol increased the expression levels of LPS-induced fat synthesis genes (PSMA5, SCD, FASN and SREBP1). These findings suggest that β-sitosterol may have the potential to alleviate the inhibitory effect of LPS on fat synthesis-related genes through the HIF-1α/mTOR signaling pathway. Therefore, β-sitosterol could be a promising strategy to prevent the reduction in milk fat synthesis-related gene expression caused by mammary inflammation.

## 4. Materials and Methods 

### 4.1. Cell Culture

The bovine mammary epithelial cell line (MAC-T cells) used in this study was kindly provided by Prof. Hong Gu Lee (Konkuk University, Seoul, South Korea). After the cells were given, the validation results were in accordance with the MAC-T cell characteristics [42]. The passage number of MAC-T cells processed in this study was between 55–57. MAC-T cells were cultivated in high-glucose Dulbecco’s Modification of Eagle’s Medium(DMEM, Hyclone, Logan, UT, USA) with extra 10% Fetal bovine serum (FBS, Gibco, Gaithersburg, MD, USA), 1% penicillin-streptomycin (Sigma-Aldrich, St. Louis, MO, USA), 5 μg/mL insulin (Sigma-Aldrich, St. Louis, MO, USA), and 1 μg/mL hydrocortisone (Sigma-Aldrich, St. Louis, MO, USA) in a 5% CO_2_ atmosphere at 37 °C.

### 4.2. Cell Treatment

β-sitosterol, with a purity of ≥95% (S24012, Yuan Ye Bio-Technology Co., Ltd., Shanghai, China), was prepared as a stock solution in anhydrous ethanol at a concentration of 40 mmol/mL and stored at −20 °C. The working solutions were further diluted in DMEM to obtain various concentrations: 0.01, 0.1, 1, 5, 10, 20, 30, and 40 µM. LPS from *E. coli* 0111: B4 (LPS 25, Sigma-Aldrich, St. Louis, MO, USA) was initially dissolved in phosphate buffer solution (PBS) at 5 mg/mL to create the stock solution. This was then diluted to 1 µg/mL in DMEM to serve as the final concentration for subsequent experiments. In a previous study, 1 μg/mL LPS was screened to cause cell damage [25]. Thus, in subsequent experiments, MAC-T cells were treated with 1 µM β-sitosterol and 1 µg/mL LPS for 24 h. The experiment was divided into four groups, the control group, the β-sitosterol group, the LPS group and the LPS+β-sitosterol group. Each treatment had three independent replicates. 

### 4.3. Cell Viability Assay

The viability of cells was measured by the CCK-8 kit (Cell counting kit-8, Meilunbio, Dalian, China) according to the manufacturer’s instructions. For the cell proliferation, MAC-T cells were seeded in 96-well plates at a density of 5 × 10^3^ cells/well. Once achieving 70–80% confluence, cells were treated with varying concentrations of β-sitosterol (0.01, 0.1, 1, 5, 10, 20, 30, or 40 µM) for 24 h. Then, 10 µL of CCK-8 reagent was added to each well, and incubation continued at 37 °C for an additional 2 h. Absorbance values were subsequently recorded at 450 nm using a microplate reader (Eon, BioTek Instruments, Winooski, VT, USA).

### 4.4. Assay of Antioxidant Enzymes Activities and T-AOC

The activities of CAT, GSH, T-AOC and T-SOD in MAC-T cells were measured by using the kits according to the instructions (Nanjing Jiancheng Bioengineering Institute, Nanjing, China). For the detection, 2.5 × 10^4^ cells/well of MAC-T cells were seeded in six-well plates treated with 1 μM β-sitosterol and 1 μg/mL LPS for 24 h. During sample extraction, adherent cells and the culture medium were harvested. Cellular protein concentration was ascertained using the BCA protein assay kit (Bicinchoninic Acid Protein Assay Kit, Meilun Biologicals, Dalian, China), with the optical density (OD) read at 592 nm. Reagents were added sequentially as outlined in the kit’s guidelines.

To quantify CAT and GSH levels, cells were cultured as previously described. Adherent cells were then scraped into 1.5 mL microtubes and homogenized. From this mixture, 100 μL of the precipitating solution was centrifuged at 250× *g* for 10 min. The supernatant was then decanted for analysis. Finally, the OD values were recorded at 405 nm. 

To determine the T-AOC content, samples were taken from the cell culture medium. After assessing the protein concentration, reagents were added based on the kit’s protocol. The mixture was then incubated at room temperature for 3–5 min, followed by absorbance measurement at 593 nm. T-SOD levels were quantified using a similar cell culture method as previously described. The mixture was incubated at room temperature for 10 min, and absorbance was then recorded at 550 nm.

### 4.5. Measurement of ROS Production

ROS was detected using the 2′,7′-dichlorodihydrofluorescein diacetate (DCFH-DA). Briefly, DCFH-DA from the ROS assay kit (Meilunbio, Dalian, China) was diluted to a final concentration of 10 µg/mL with serum-free medium. MAC-T cells were seeded in six-well plates, when the cells reached 70–80% confluence, treated with either β-sitosterol or LPS for 24 h. The MAC-T cells were washed with 1×PBS thoroughly for one time, followed by adding 2 mL of diluted DCFH-DA working buffer and incubated at 37 °C for 30 min, then the cells were washed three times with serum-free medium to remove the unbound dye. Fluorescence intensity was measured by using a BioTek CYTATION 5 imager (Winooski, VT, USA) and analyzed by Image J v1.8.

### 4.6. Cell Apoptosis Assay

MAC-T cells were stained using Annexin V-FITC/PI Apoptosis Detection Kit (Meron Bio, Dalian, China) to determine the apoptosis rate. MAC-T cells cultured in six-well plates were collected in 15 mL tubes and centrifuged at 1000× *g* for 5 min, discard the supernatant. The cells ware washed twice with 1× PBS and resuspended with 1× binding buffer. 100 µL cell suspension was pipetted into new 1.5 mL micro tubes, 5 µL Annexin V-FITC and 5 µL PI were added, incubated for 15 min at room temperature shielded from light, then mixed 400 µL 1× binding buffer. Detected by flow cytometry CytoFLEX BA32233 (Beckman Coulter Biotechnology, SuZhou, China) (at least 1 × 10^4^ cells per sample), and the distribution was analyzed by CytExpert 2.4 software. The experiments were performed in triplicate.

### 4.7. Total RNA Extraction and Quantitative Real-Time PCR

Total RNA was isolated from MAC-T cells cultured in six-well plates using TRIzol reagent (Thermo Scientific, Waltham, MA, USA) according to the instructions of the reagent manufacturer. The concentration and purity of total RNA samples were determined with a NanoDrop 2000 spectrophotometer (Thermo Scientific, Waltham, MA, USA). The cDNA was synthesized from the total RNA according to the instructions of HiFi Script cDNA Synthesis Kit (CWBIO, Beijing, China) and ECO Gene amplification instrument (BIOER, HangZhou, China). The synthesized 2.0 µL cDNA, 10 µL UltraSYBR mixture (CWBIO, Beijing, China), 0.4 µL upstream primer, 0.4 µL downstream primer, and 7.2 µL ddH_2_O were then mixed in an eight-strip tubes. The quantitative real-time PCR was performed by the following steps, 95 °C for 10 min, 95 °C for 10 s, 60 °C for 30 s and at 72 °C for 32 s by 40 cycles. Quantitative fluorescence values were calculated relative mRNA levels of genes by using the 2^−∆∆CT^ method. Table 1 listed the primers sequences used in this study.

### 4.8. Western Blotting

Total protein from MAC-T cells was extracted with RIPA buffer (Sigma-Aldrich, St. Louis, MO, USA), and the concentration of extracted protein was determined by BCA kit. The protein samples were mixed with the loading buffer and heated at 95 °C for 10 min. According to the target protein size, appropriate protein separation gels were configured for sodium dodecyl sulfate–polyacrylamide gel electrophoresis (SDS-PAGE), and 25 μg of protein samples were loaded into each well. Proteins were subsequently transferred to polyvinylidene difluoride (PVDF) membranes. These membranes were then blocked with 5% BSA for 1 h at room temperature and subsequently incubated with primary antibodies overnight at 4 °C. Following primary antibody incubation, the PVDF membranes were washed four times with 1× Tris-buffered saline-tween (TBST) for 10 min per wash. They were then exposed to the appropriate secondary antibodies for 1 h at room temperature. Protein bands were visualized on a chemiluminescence imager using the ECL Western blotting substrate (Meilunbio, Dalian, China). Protein levels were quantified using Image J 1.53 analysis software, and each protein was normalized against β-actin levels. Details of antibodies are shown in Table 2.

### 4.9. Statistical Analyses

Data analysis was conducted using SPSS 26.0 software (SPSS Inc., Chicago, IL, USA). All experiments were repeated with three independent replicates. Differences among treatment groups were determined using one-way ANOVA. For post hoc comparisons, the Least Significant Difference (LSD) test and Duncan’s Multiple Range Test were utilized. Data were presented as the mean ± SEM. A *p*-value of less than 0.05 was considered as statistically significant. 

## 5. Conclusions

**Figure 7:**
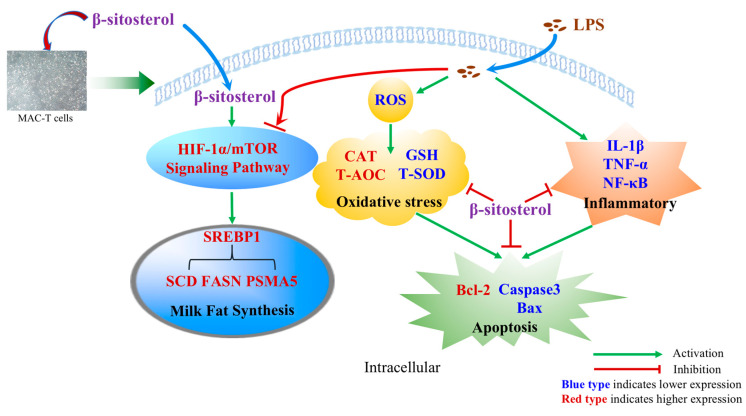
Schematic diagram showed that β-sitosterol reduced LPS-induced oxidative stress and inflammation, while increasing the expression of anti-apoptotic proteins and activating the HIF-1α/mTOR signaling pathway to inhibit apoptosis and improve lipid synthesis-related gene expression.

This study suggests that β-sitosterol may have a protective effect against LPS-induced injury in MAC-T cells. It reduces oxidative stress by decreasing ROS accumulation and restoring antioxidant enzyme activity, as well as suppresses inflammation by inhibiting the NF-κB pathway and mitigates apoptosis by inhibiting the Caspase/Bcl-2 pathway and activating the mTOR/HIF-1α signaling pathway. Additionally, β-sitosterol improves the expression of genes involved in milk fat synthesis, ultimately ameliorating LPS-induced inflammation in cells (Figure 7). While β-sitosterol shows promise as an alternative to antibiotics for alleviating mastitis, further research is necessary to explore its specific mechanism of action and practical applications in production.

## Figures and Tables

**Table 1:** Gene information and polymerase chain reaction (PCR) primer sequences.

Gene Name	Accession Number	Primers Sequence	Product Size, bp
β-actin	NM_173979.3	F: 5′-CCCTGGAGAAGAGCTACGAG-3′ R: 5′-GTAGTTTCGTGAATGCCGCAG-3′	130
IL-1β	NM_174093.1	F: 5′- GTCCTCCGACGAGTTTCTGT-3′ R: 5′- AGAGCCTTCAGCACACATGG -3′	111
TNF-α	NM_173966.3	F: 5′-AAGCCTCAAGTAACAAGCCGGTAG-3′ R: 5′-TCACACCGTTGGCCATGAG-3′	108
NF-κB (p65)	NM_001080242.2	F: 5′-ACCTGGGGATCCAGTGTGTA-3′ R: 5′-ACGGCATTCAGGTCGTAGT-3′	127
Caspase 3	NM_001077840.1	F: 5′-CGAGGCACAGAACTGGACTG-3′ R: 5′-ATGCGTACAAGAAGTCTGCCT-3′	100
Bax	XM_015458140.2	F: 5′-GCTCTGAGAGATCATGAAGAC-3′ R: 5′- CAATTCATCTCCGATGCGCT -3′	166
HIF-1α	NM_174339.3	F: 5′-TTCCATCTCCTCCCCACGTA-3′ R: 5′-AGGCTGTCCGACTTCCAGTA-3′	81
mTOR	XM_002694043.6	F: 5′-CGAAGAACCAATTATACCCGC-3′ R: 5′-CATAGCAACCTCAAAGCAGTCC-3′	153
FASN	NM_001012669.1	F: 5′-GACCTGGGAGGAGTGTAAGC-3′ R:5′-GCGATAGCGTCCATGAAGTA-3′	198
SREBP1	NM_001113302.1	F: 5′-CGCTCTTCCATCAATGACA-3′ R:5′-TTCAGCGATTTGCTTTTGTG-3′	188

**Table 2:** Antibody information and dilutions in this study.

Antibodies Name	Diluted Multiples	Accession Number	Reagent Company
Rabbit anti-β-actin polyclonal antibody	1:2000	bs-0061R	Bioss
Rabbit anti- NF-κB (p65) polyclonal antibody	1:2000	BSP4135	Bioworld
Rabbit anti-Caspase 3 polyclonal antibody	1:2000	bs-0081R	Bioss
Rabbit anti-Bax polyclonal antibody	1:2000	bs-0127R	Bioss
Rabbit anti-Bcl-2 polyclonal antibody	1:2000	12789-1-AP	Proteintech
Rabbit anti-HIF-1α polyclonal antibody	1:500	bs-0737R	Bioss
Rabbit anti-mTOR polyclonal antibody	1:2000	28273-1-AP	Proteintech
Rabbit anti-phospho-mTOR polyclonal antibody	1:2000	67778-1-lg	Proteintech
Rabbit anti- SCD polyclonal antibody	1:1000	bs-3787R	Bioss
Mouse anti-PSMA5 polyclonal antibody	1:1000	bsm-51520M	Bioss
Rabbit anti-LPL polyclonal antibody	1:1000	bs-1973R	Bioss
Goat anti-rabbit IgG antibody	1:20,000	bs-40295G-HRO	Bioss
Goat anti-mouse IgG antibody	1:20,000	bs-40296G-HRP	Bioss

## Data Availability

Data in this study are available from the corresponding authors upon request.
